# Characterization of cerebral aneurysms using 4D FLOW MRI

**DOI:** 10.1186/1532-429X-14-S1-W2

**Published:** 2012-02-01

**Authors:** Susanne Schnell, Sameer A Ansari, Parmede Vakil, Michael Hurley, James Carr, Hunt Batjer, Bernard R Bendok, Timothy J Carroll, Michael Markl

**Affiliations:** 1 Dept. of Radiology, Northwestern University, Feinberg Medical School, Chicago, IL, USA; 2 Dept. of Neurological Surgery, Northwestern University, Feinberg Medical School, Chicago, IL, USA

## Summary

Six patients with large or giant cerebral aneurysms were examined with 4D-Flow MRI and analyzed regarding 3D flow patterns and aneurysm wall shear stress (WSS). Two distinct groups of aneurysms were identified with fast swirling flow versus slow flow and significantly different WSS patterns, correlating with aneurysm morphology.

## Background

Cerebral aneurysms are diverse and life threatening conditions. They typically develop at the major bifurcation sites of the intracranial vessels. In general, increasing size has been linked to higher rupture risk but specific data concerning which lesions will grow or de-stabilize is lacking. In patients with large and giant cerebral aneurysms, 4D-Flow MRI was employed to characterize hemodynamics and WSS patterns and their association with aneurysm location, shape and size.

## Methods

**Figure 1:**
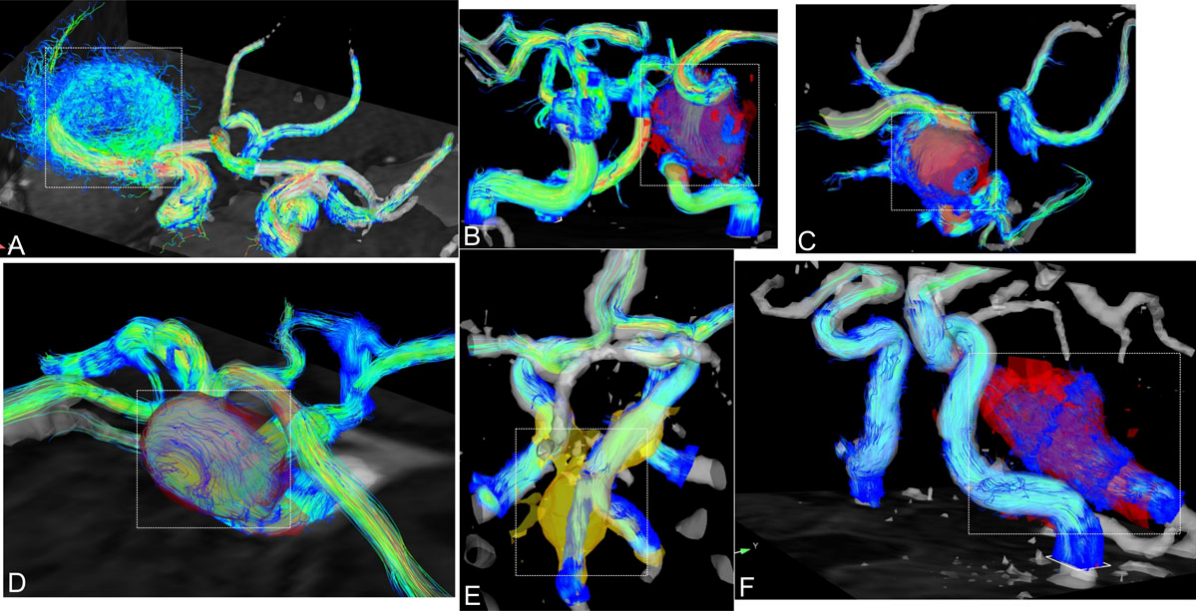
Images A to D show aneurysms of group 1 (fast flow with clear flow channels) with spherical shape located at segment C7 of ICA or M1 of MCA. Images E and F show the two fusiform aneurysms of group 2 with slow and swirling flow located at the junction of the vertebral arteries or in the basilar artery. The dashed white boxes indicate the location of the aneurysms.

**Figure 2:**
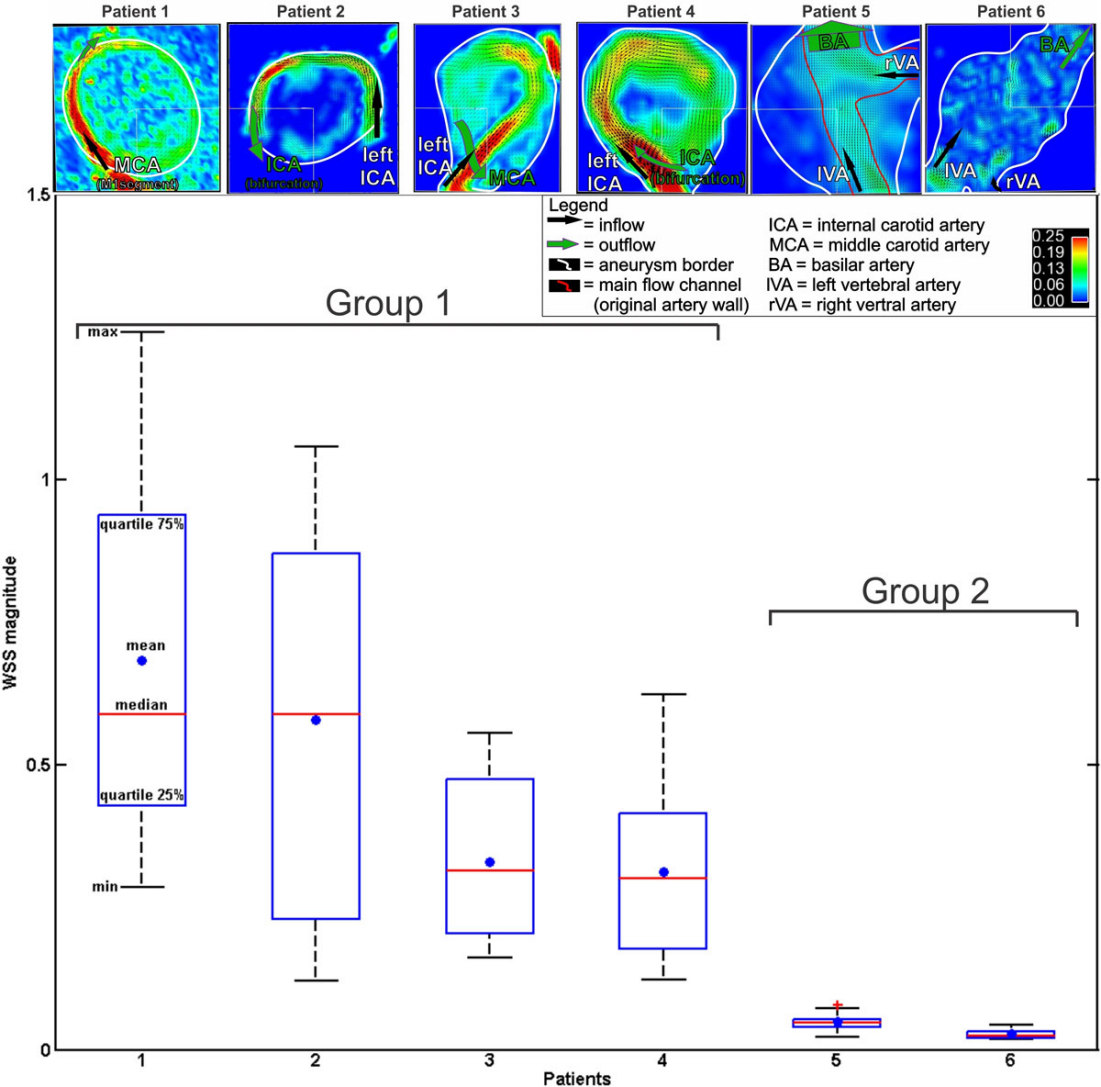
Box Plot of WSS for 12 equally distributed points along the border of the outlined plane through the aneurysm. The WSS was averaged over all cardiac cycles. The top row shows the chosen cut-planes through the aneurysms. A paired t-test showed that group 1 and group 2 have different means, meaning they are drawn from different distributions. The significance was below 0.01 (2-tailed).

Six patients (4 females, 2 males, mean age 56.6 ±14.8) with large or giant cerebral aneurysms (mean largest dimension = 2.6 +/-0.9, range = 1.4 - 4.2 mm) were studied. Aneurysms were located near the ICA bifurcation (n=4) with a saccular/spherical morphology or the basilar artery (n=2) with a fusiform morphology and examined using 4D flow MRI (3T TRIO & 1.5T Avanto, Siemens, Germany, spatial resolution = 0.99-1.8 mm x 0.78-1.46 mm x 1.2-1.4 mm, temporal resolution = 5.5-6 ms, 3-directional velocity encoding with venc = 70-80). Data were analyzed with in-house Matlab-based tools (Bock et al., Proc ISMRM 2007) and 3D blood flow visualization software (ENSIGHT, CEI, USA). Intra aneurysmal flow was visualized using time-resolved pathlines (Figure 1) and vector graphs mapped onto a 2D plane through the center of the aneurysm (Figure 2). The WSS pattern along the aneurysm wall was calculated by cubic spline interpolation of the velocity gradient along the aneurysm contour as described previously (Stalder et al., MRM2007).

## Results

The combination of 3D spatial encoding and 3-directional velocity encoding allowed for the 3D visualization of complex intra-cranial flow patterns (Figure 1). All aneurysms could be well segmented using the velocity data combined with magnitude data. As shown in Figure 2, flow patterns in the six aneurysms were classified in two morphological groups. Narrow high-flow channels along the aneurysm wall in combination with large central low flow regions were identified in saccular/spherical aneurysms of the anterior circulation (ICA or MCA group 1, n=4). In contrast, slow flow with less defined flow channels were noted in fusiform aneurysms (VA or BA group 2, n=2) (Figure 2, top row). The distribution of WSS along the aneurysm wall (white lines, Figure 2) was significantly more heterogeneous and increased for group 1 compared to group 2 (paired t-test after verification of normal distribution, 0.63+/-0.33 vs. 0.038+/-0.016, Sig(2-tailed)<0.01).

## Conclusions

The findings of this feasibility study show the potential of 4D flow MRI to identified differences in flow characteristics and WSS patterns in two intracranial aneurysm morphology groups. Future longitudinal studies based on the measurement, analysis and visualization of cerebral aneurysms using 4D-Flow MRI have the potential to correlate disease progression with regional hemodynamics; and may thus, improve risk stratification and interventional or surgical treatment planning.

## Funding

DFG (German Research Foundation) SCHN 1170/2-1.

